# BJPsych Bulletin In Memoriam List December 2025

**DOI:** 10.1192/bjb.2025.10176

**Published:** 2025-12

Annear, John Marshall, London, UK, FRCPsych 1995

Batchelor, David Hamilton, Hungerford, UK, Mental Health Associate 1993

Bell, Cara, Barnsley, UK, MRCPsych 2018

Bell, David Samuel, Cremorne, Australia, FRCPsych 1991

Bendi, Nagendra, Norwich, UK, MRCPsych 2013

Bernheim, Jacques Bernard, Geneve, Switzerland, FRCPsych 1987

Boyd, Cameron Edward, Liverpool, UK, FRCPsych 1995

Bownes, Ian Thomas, Bangor, UK, MRCPsych 1987

Browne, Michael William, London, UK, FRCPsych 1989

Brooker, Bertram Keir, Truro, UK, MRCPsych 1964

Bryant, Francis Fabian, Paddington, Australia, MRCPsych 1967

Churcher-Brown, Christopher John, Waterlooville, UK, FRCPsych 1999

Cordingly, John, London, UK, FRCPsych 1989

Davies, Martin Hugh, Birmingham, UK, FRCPsych 1981

Davison, Kenneth, Newcastle upon Tyne, UK, FRCPsych 1972

Eastwood, Michael Robin, Yelverton, UK, FRCPsych 1977

Faulk, Malcolm, Winchester, UK, FRCPsych 1979

Fenwick, Peter Brooke Cadogan, London, UK, FRCPsych 1986

Fernando, Sumantra Jayanandana Mohandas, London, UK, FRCPsych 1986

Foyle, Marjory Florence, London, UK, FRCPsych 1980

Franklin, Robert Adrian, Christchurch, UK, FRCPsych 1984

Gayford, John, Horley, UK, FRCPsych 1986

Hession, Michael Anthony, Hereford, UK, FRCPsych 1994

Hodgson, Gail Iona, Westbury-on-Severn, UK, MRCPsych 1988

Hussain, Sharafat, Newcastle upon Tyne, UK, FRCPsych 2004

Jansen, Marlies Rosalie Gabrielle, Alderton, UK, MRCPsych 2002

Jayaram, Ramaswamy, Ipswich, UK, MRCPsych 2008

Karki, Chuda Bahadur, Chelmsford, UK, FRCPsych 2000

Kershaw, Peter Whaley, Bearsden, UK, FRCPsych 1981

Levy, Raymond, London, UK, FRCPsych 1973

Lindsay, Mary Katherine Martin, Tring, UK, FRCPsych 1979

Little, Margaret Joyce, Glasgow, UK, MRCPsych 1977

Lucas, Christopher, Reading, UK, MRCPsych 2021

Lund, Charles Ames, Newcastle upon Tyne, UK, FRCPsych 1987

Melichar, Jan Krzysztof, Bristol, UK, FRCPsych 2015

Neeleman, Jan, Draguignan, France, MRCPsych 1992

O’Brien, Laurence Stephen, Manchester, UK, FRCPsych 2001

O’Gorman, Ethna Catherine, Saintfield, UK, FRCPsych 1987

Parkes, Colin Murray, Rickmansworth, UK, FRCPsych 1975

Parkianathan, Rajeeve, Norwich, UK, MRCPsych 2012

Perry, David William, Solihull, UK, MRCPsych 1994

Phillips, John Ernest, Norwich, UK, FRCPsych 1987

Rankin, Donald Watson, Kirkcaldy, UK, FRCPsych 1990

Roberts, Julian Mervyn, Ilkley, UK, FRCPsych 1972

Russell, John Anderson Oliver, Bristol, UK, FRCPsych 1985

Salmons, Pauline Hilary, Macclesfield, UK, FRCPsych 1987

Samad, Abdus, Burwood Heights, Australia, MRCPsych 1964

Sanderson, Alan Lindsay, London, UK, MRCPsych 1965

Singh, Gurpreet, Branston, UK, Specialist Associate 2013

Spencer, David John, Goole, UK, FRCPsych 1987

Steen, Leslie, Manchester, UK, MRCPsych 1972

Stevenson, Pauline Frances, Newent, UK, MRCPsych 1976

Swift, Joseph Louis, Reading, UK, FRCPsych 1979

Toms, Rhinedd Margaret, Norwich, UK, FRCPsych 1993

Wardill, Lesley Forster, Saffron Walden, UK, MRCPsych 1982

Wawman, Ronald John, Okehampton, UK, FRCPsych 1980

Weller, Malcolm, London, UK, FRCPsych 1987

White, Ruth Elizabeth Bevis, Presteigne, UK, MRCPsych 1988

Wilson, Alastair, Bearsden, UK, FRCPsych 2011

